# Enhancing HIV pre-exposure prophylaxis outcomes among Kenyan adolescent girls and young women with a novel pharmacy-based PrEP delivery platform: protocol for a cluster-randomized controlled trial

**DOI:** 10.1186/s13063-024-08206-6

**Published:** 2024-06-19

**Authors:** Harison K. Lagat, Jillian Pintye, Elizabeth Harrington, Samantha Houck, Zachary Kwena, Meena Lenn, Felix Mogaka, Vincent Momanyi, Melissa Mugambi, Bernard Nyerere, Josephine Odoyo, Victor Omollo, Katrina F. Ortblad, Greshon Rota, Monisha Sharma, Elizabeth A. Bukusi

**Affiliations:** 1 University of Washington, Seattle, WA USA; 2 Kenya Medical Research Institute, Nairobi, Kenya; 3 Fred Hutchinson Cancer Center, Seattle, WA USA

**Keywords:** AGYW, HIV prevention, Nurse navigator, Pharmacy-based delivery, Pre-exposure prophylaxis, PrEP adherence

## Abstract

**Background:**

In Kenya, 65% of sexually active unmarried women use modern contraceptives, a population at increased risk of HIV acquisition compared to other populations. Anchoring HIV prevention services, including pre-exposure prophylaxis (PrEP), to trusted contraceptive delivery settings offers opportunities to efficiently reach this important population. In Kenya, almost half (40%) of women accessing contraception services do so outside traditional healthcare facilities, such as retail pharmacies. Thus, integrating PrEP services into retail pharmacies may increase options for reaching adolescent girls and young women (AGYW) who could benefit from PrEP. Efforts are underway to define care pathways for pharmacy-delivered PrEP services in Kenya, including unsupported and supported models with nurse navigators.

**Methods:**

The AGYW Pharmacy PrEP study is an unblinded 2-arm cluster-randomized controlled trial in Kisumu, Kenya. The objective is to determine the effect that unsupported versus supported pharmacy-delivered PrEP services has on PrEP initiation, persistence, and adherence among AGYW seeking contraception. Twenty retail pharmacies offering pharmacy provider-led PrEP delivery will be randomized 1:1 to either receive or not receive a nurse navigator to support PrEP delivery. Eligible AGYW (*n* = 1900 total, *n* = 950/arm) will be ≥ 15 years old, purchasing a method of contraception at the pharmacy. Trained pharmacy provider will offer eligible AGYW either daily oral PrEP or the monthly DPV vaginal ring. The primary trial outcomes are PrEP initiation (use of PrEP at 1 month), persistence (use of PrEP at 10 months), and adherence (quantified by levels of TFV or DPV in hair samples). Additionally, several secondary (STI incidence, PrEP method selection, predictors of PrEP adherence) and exploratory outcomes (HIV incidence, quality of care, contraceptive method mix) will be explored.

**Discussion:**

We hypothesize pharmacy-delivered PrEP services supported with nurse navigator, versus delivered by pharmacy providers alone, will improve PrEP outcomes among AGYW seeking contraception. Our results will help policy makers better understand how to potentially implement this novel differentiated service model for PrEP and prime pharmacies for the delivery of new PrEP agents in the pipeline (e.g., long-acting injectables and multi-purpose technologies). The study was initiated on May 13, 2023, and is expected to be completed by February 2025.

**Trial Registration:**

ClinicalTrials.gov (NCT05467306), with registration on July 20, 2022.

## Introduction

### Background and rationale 

In Kenya, 65% of unmarried sexually active women use modern contraceptives [1] and may benefit from engagement in HIV prevention services, including pre-exposure prophylaxis (PrEP) with daily oral pills or the monthly dapivirine vaginal ring (DPV-VR). Anchoring PrEP service delivery to settings women already trust and routinely access for contraception may help reach adolescent girls and young women (AGYW), who are at risk of HIV acquisition in Kenya. In Kenya, almost all PrEP service delivery occurs through public health clinics; however, almost half (40%) of women access contraception outside clinics including at retail pharmacies [1]. Thus, implementing novel models of PrEP delivery that operate outside traditional clinic settings to reach AGYW who do not frequently attend public clinics can help maximize the potential impact of PrEP[2].

Delivery of PrEP at retail pharmacies is a promising strategy to expand PrEP coverage. Compared to public clinics, retail pharmacies are often more accessible, as they are ubiquitous and typically located in high-traffic areas [3–5]. Additionally, services delivered at pharmacies may be more private and confidential than those delivered at public clinics, as individuals often visit pharmacies for a variety of reasons unrelated to their healthcare needs [6–8]. Pharmacies may also be more responsive to client demands compared to public clinics, as they are typically self-sustained via fee-for-service care and thus have a vested interest in satisfying customers for sustained and repeat client engagements [9–12]. Several formative qualitative studies and pilot studies on pharmacy-delivered PrEP services, mainly conducted in Kenya and South Africa, have found that this novel model of PrEP delivery is feasible, acceptable, and preferred by pharmacy clients, including AGYW [13–16].

One pharmacy-delivered PrEP services model tested in these pilots entailed a nurse navigator stationed at a retail pharmacy who facilitated PrEP delivery for AGYW seeking contraception [15, 16]. In this pilot, Kenyan AGYW (*n* = 235) offered daily oral PrEP at two retail pharmacies frequently initiated (85%), planned to continue using (68%), and were willing to pay for PrEP services (69%), even when these were available for free at public clinics—suggesting sustainability of this approach [15]. Participants cited convenience, short wait times, less stigma, and high-quality counseling on PrEP and family planning services by the nurse navigators at the pharmacies as their reasons for preferring pharmacy- versus clinic-based PrEP services [17, 18].

In this cluster-randomized controlled trial (cRCT) in Kenya, we will determine the additional benefit (i.e., effect) of stationing a nurse navigator at retail pharmacies to support pharmacy-delivered PrEP services (versus pharmacy-delivered PrEP services without a nurse navigator) on PrEP initiation, persistence, and adherence among AGYW seeking contraception.

### Objectives 

We aim to determine the effect of nurse navigators on PrEP initiation, persistence, and adherence among HIV-negative AGYW (age 15–24 years) seeking contraception by comparing pharmacy-based PrEP delivery models with and without nurse navigators.

### Trial design 

This is a two-arm, unblinded, cluster-randomized controlled trial in which 20 retail pharmacies offering standard PrEP delivery are randomized 1:1 to either receive or not receive a nurse navigator to support PrEP delivery for AGYW.

## Methods: participants, interventions, and outcomes

### Study setting 

**Fig. 1:**
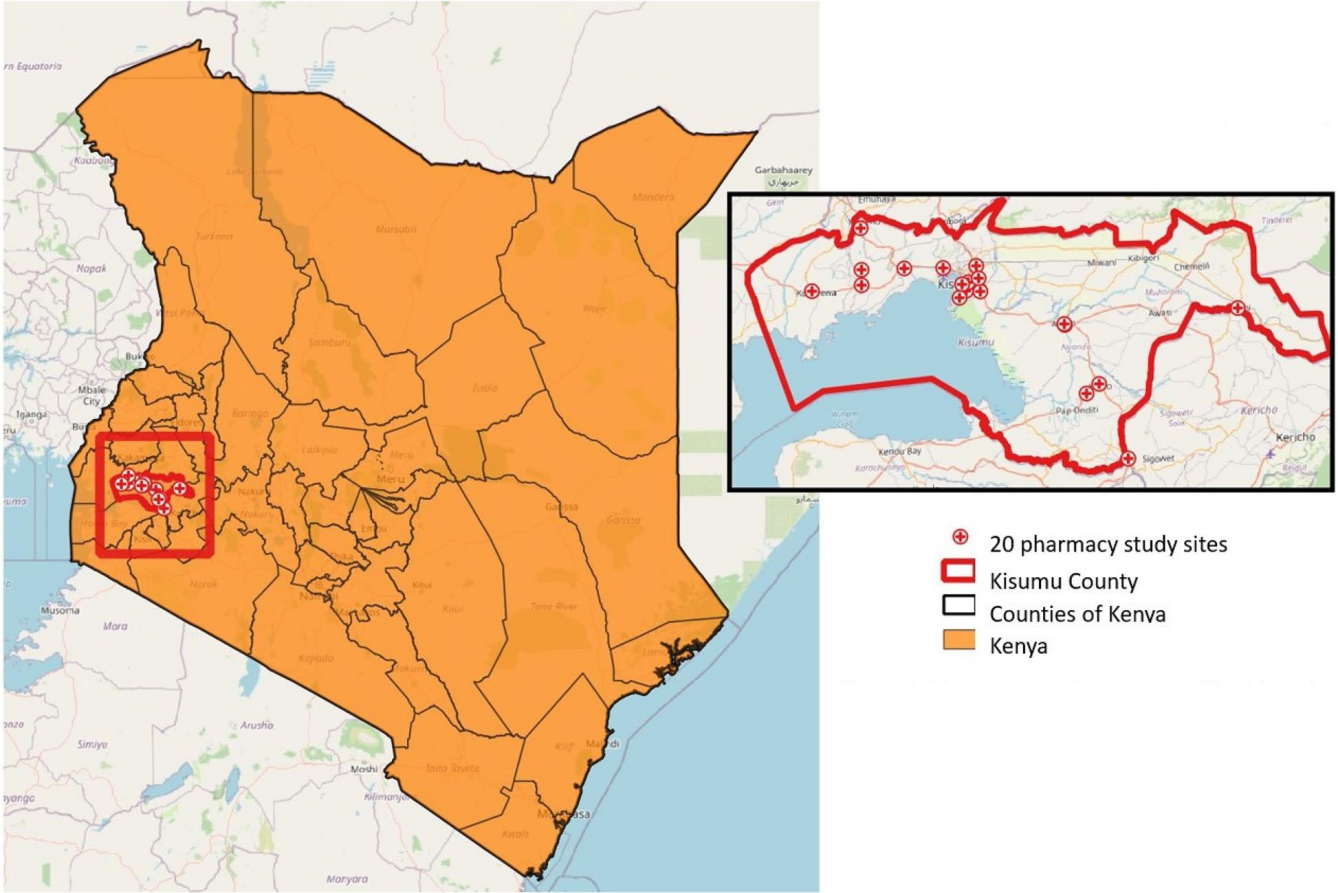
Distribution of study sites in Kisumu County, Kenya [20]

The study will be conducted in Kisumu County, Kenya, where HIV prevalence is 21% among cisgender women [17, 18]. We conducted a landscape analysis of the 119 retail pharmacies in Kisumu to gather information on the types of customers who attend, services offered (e.g., HIV testing), and products purchased (e.g., contraceptive methods) at pharmacies as well as the availability of a separate, private consultation room. Based on this assessment, we selected 20 retail pharmacies that met study criteria: were willing to provide PrEP services per the national guidelines [19], had a separate consultation room for HIV testing and PrEP counseling, and provided a variety of contraceptives to AGYW clients, including emergency contraception, oral contraceptive pills, injectables, implants, and condoms (Fig. 1).

### Eligibility criteria 

All participants are screened for eligibility and asked to provide oral and written informed consent before study participation. At screening, eligibility information is obtained. The results from the screening assessments are reviewed for the inclusion/exclusion criteria described below:

Inclusion

- Female gender
- Seeking contraception (EC, OCP, injectables, implants, and condoms) from the retail pharmacy site
- Age between > 14 and < 25 years old
- Willingness to receive PrEP screening per national guidelines including HIV testing
- Not currently taking PrEP
- Able and willing to provide informed consent for participation

Exclusion

- Male gender
- Not seeking contraception (EC, OCP, injectables, implants, and condoms) from the retail pharmacy site
- Age < 15 or > 24 years old
- Not willing to receive PrEP screening per national guidelines or not willing to receive HIV testing
- Currently taking PrEP
- Not able or willing to provide informed consent for participation

### Who will take informed consent? 

All potential participants are screened for eligibility and then asked to provide oral and written informed consent by a trained study team member before study participation. The parental consent will be waived for AGYW aged 15–17 years. This waiver is based on the recommendations of the Ministry of Health’s Guidelines for Conducting Adolescent HIV Sexual and Reproductive Health Research in Kenya (2015) [21]. According to the Kenya Ministry of Health guidelines, adolescents are able to provide consent for HIV and STIs testing and treatment in Kenya and therefore parental consent can be waived for HIV/STI related studies.

### Additional consent provisions for collection and use of participant data and biological specimens 

The study provides information on specimen collection during enrollment. Specimen collection is not performed unless informed signing of the study consent form is confirmed. In addition to consenting to participate in the study, participants opt in or out of agreeing to store samples at the Kisumu site and the University of Washington for future research into HIV, HIV-related diseases, STIs, and other infectious diseases.

## Interventions

### Explanation for the choice of comparators 

**Fig. 2:**
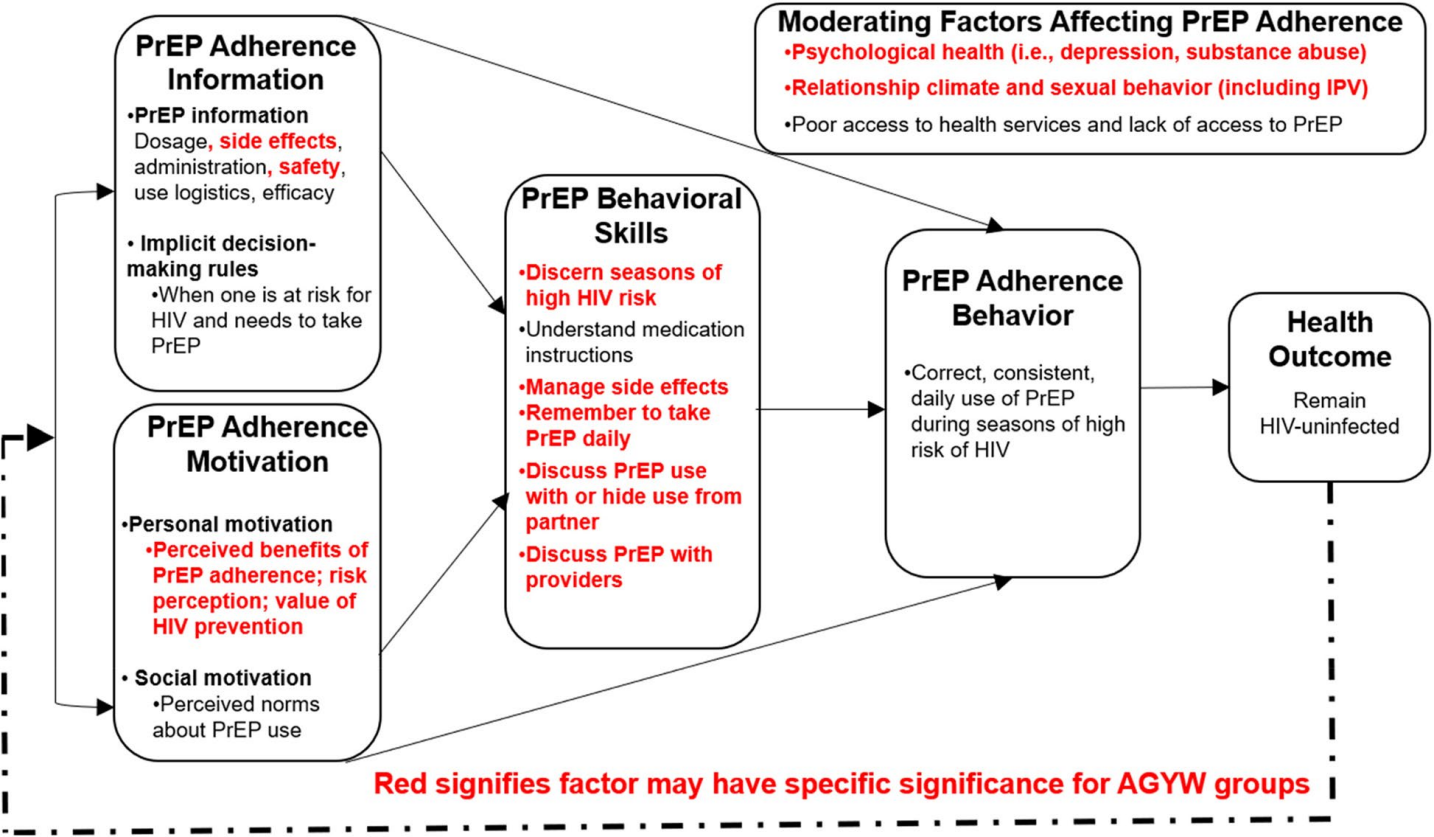
Information-motivation-behavioral skills model for PrEP adherence (adapted from Fisher 2006 and Ferrer et al. 2010). AGYW, adolescent girls and young women; HIV, human immunodeficiency virus; PrEP, pre-exposure prophylaxis

An information-motivation-behavioral skills (IMB)-guided approach informed the development of the nurse navigator model for pharmacy-based PrEP delivery tailored to AGYW, using quantitative and qualitative data from the pilot (Fig. 2) [15, 22]. The nurse navigator model is designed to target domains of the IMB (PrEP adherence information, PrEP adherence motivation, PrEP behavioral skills, PrEP adherence behavior and health outcome). Nurse navigators expand PrEP support for AGYW and provide opportunities for dialogue beyond PrEP, enabling AGYW to receive counseling on contraception, relationships, and other concerns across IMB domains, which may facilitate PrEP use.

To our knowledge, this pilot was the first evaluation of a pharmacy PrEP delivery tailored to AGYW and our nurse navigator model addressed issues specific to AGYW in the context of contraception. Our pilot results provided a signal to formally test nurse navigators as a strategy to improve PrEP outcomes among AGYW in a rigorous cluster randomized clinical trial with a comparison group of standard pharmacy-based PrEP without nurse navigators.

### Intervention description 

**Fig. 3:**
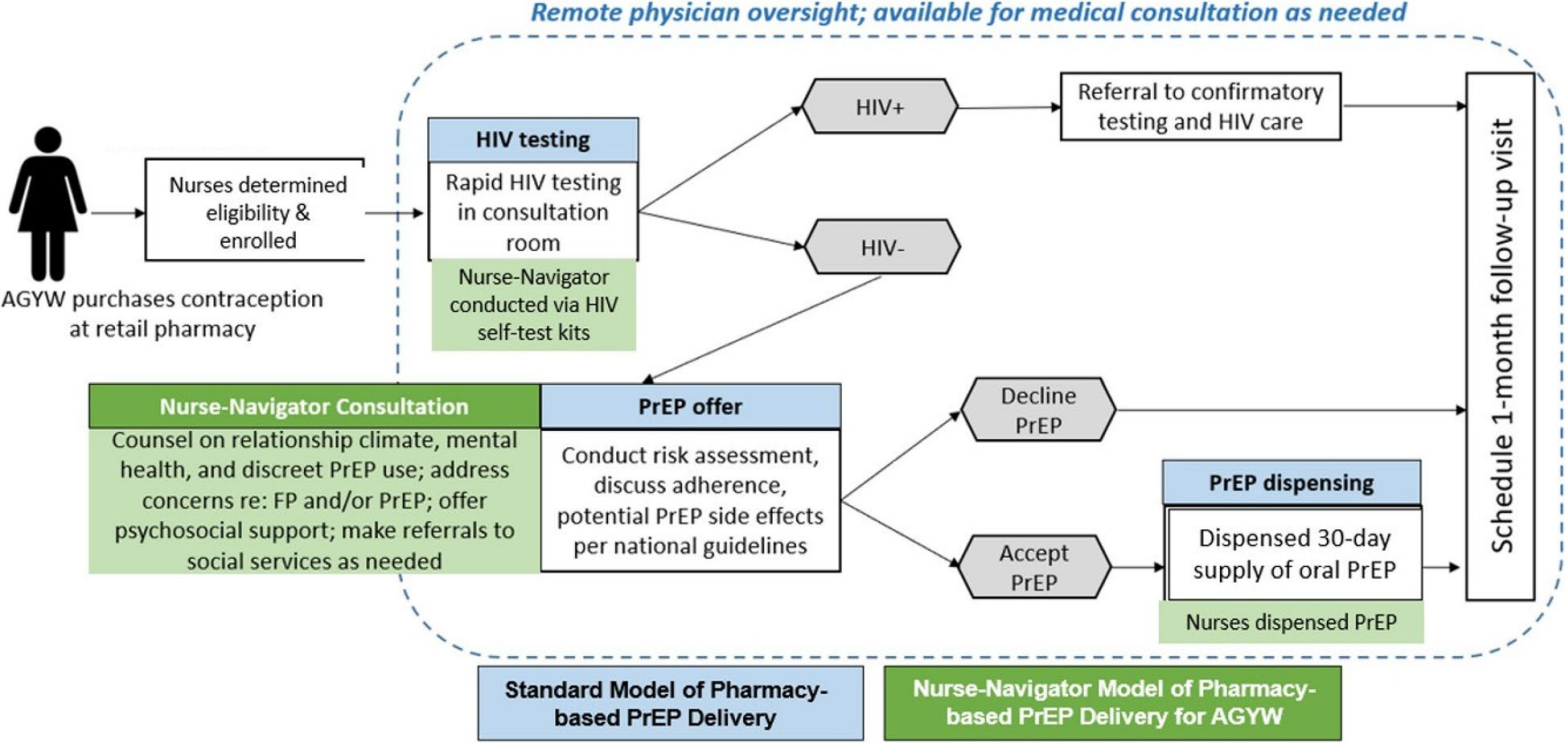
PrEP pathway in randomization arms. AGYW, adolescent girls and young women; HIV, human immunodeficiency virus; PrEP, pre-exposure prophylaxis [23]

In both arms, AGYW receive standard PrEP services at the pharmacy, which entail a pharmacy provider using a prescribing checklist to identify individuals with risk for HIV acquisition and at low risk of any drug contraindications, counsel them on PrEP adherence (per national guidelines), test for HIV, and prescribe/dispense PrEP with remote clinician oversight. All participants offered PrEP self-select either daily oral PrEP pills or the DPV-VR. All participants are provided with a study phone number to call free of charge for clarifying questions or concerns with PrEP use (Fig. 3).

Pharmacies randomized to the nurse navigator model receive tailored support counseling in addition to standard PrEP services. Support counseling by nurse navigators provides educational messaging tailored to AGYW and actionable advice targeting PrEP persistence and adherence and/or FP topics and addresses participants’ questions related to the content at each study visit. Counseling includes adherence encouragement (IMB domain: motivation), PrEP efficacy and safety (IMB domain: information), self-efficacy for prevention of HIV, support for potential PrEP side effects, behavioral skills (tips for remembering PrEP medications, IMB domain: behavioral skills), and strategies for remembering PrEP refill schedules (Fig. 1). All participants receive PrEP adherence counseling per Kenyan national guidelines.

### Criteria for discontinuing or modifying allocated interventions 

N/A. Study participants can decline or discontinue PrEP at any time, and PrEP use is not a criteria for study participation. The nurse navigator intervention is allocated at the pharmacy-level and therefore cannot be discontinued or modified.

### Strategies to improve adherence to interventions 

N/A. The nurse navigator intervention is allocation at the pharmacy-level and therefore individual study participant adherence to the intervention is not required.

### Relevant concomitant care permitted or prohibited during the trial 

The study participants are permitted to seek services within the study sites or in any other healthcare facility without being penalized whatsoever.

### Provisions for post-trial care 

The study will prioritize the safety of the study participants as per ethical requirements. If a participant requires medical care that the study cannot provide, the study clinicians will refer participants to the appropriate services or organizations that can provide care. In cases of adverse events whether related to study participation or not, we will refer them for proper management. If a participant seroconverts while on PrEP, we will ensure they are referred to the HIV care clinic for confirmatory testing and linkage to care as per the national guidelines. The study provides comprehensive PrEP training to the pharmacy providers and nurses delivering PrEP to ensure they can continue to provide PrEP services post-trial.

### Outcomes 

Our primary outcome is PrEP initiation among AGYW seeking contraception at retail pharmacies at enrollment and evidence of self-reported use at 1-month post-use (Table 1). Our second primary outcome is PrEP persistence at 10 months among AGYW who initiated PrEP. Our third primary outcome will be PrEP adherence defined as either TFV or DPV hair levels (depending on PrEP method) at 10 months.

**Table 1:** Primary, secondary, and exploratory outcomes of the trial

Outcome		Definition
Primary	PrEP initiation	Binary endpoints (yes, no) of self-reported use of oral PrEP or DPV-VR at 1 month after acceptance at enrollment. Self-reported PrEP use will be considered initiation. If a participant declined or reported not using PrEP after acceptance, the participant will be considered non-initiated
	PrEP persistence	Binary endpoints (yes, no) of self-reported use of oral PrEP or DPV-VR at 10-month after acceptance at enrollment. Self-reported PrEP use will be considered persistence. If a participant declined PrEP or reported not using PrEP after acceptance or discontinued PrEP before 10 months will be considered non-persistent [24]
	PrEP adherence	Binary endpoints (yes, no) of detectable TDF levels in hair > 0.038 ng/mg or DPV levels > 0.0248 n/mg at 10-month visit will be considered adherent. If a participant stopped PrEP use or exited study before 10 months (i.e., no hair sample collected), the participant will be considered non-adherent
Secondary	PrEP method selection	Binary endpoints (yes, no) of self-reported DPV-VR compared to daily PrEP when offered at enrollment
	Predictors of non-adherence	Factors associated with poor adherence (< 90% adherent on PrEP or discontinuation) will include demographics, relationship/partner characteristics, psychosocial factors, socio-economic status, low health/HIV literacy, fear of disclosure
	STI incidence and cofactors	Frequency of STI (syphilis, gonorrhea, and chlamydia) detection at follow-up will be compared between randomization arms
Exploratory	HIV incidence	Frequency of HIV diagnosis at follow-up visits will be compared between randomization arms
	Quality of care	Binary endpoints (yes, no) based on MEASURE Evaluations indicators for quality of care in family planning (e.g., Did the provider ask if you were having a problem with your FP method?)
	Contraceptive method mix	Binary endpoints (yes, no) or LARC use will be compared between randomization arms and distribution of contraceptive method types

### Participant timeline 

Following written informed consent for participation, AGYW will be enrolled. Enrollment will include a detailed questionnaire, including assessment of demographic and behavioral characteristics, contraceptive history, education, marital status, income, relationship characteristics, HIV status and testing results, and clinical characteristics. Participants will be followed for 10 months, and study visits will be aligned with standard PrEP follow-up visits per national guidelines (i.e., first visit 30 days after PrEP acceptance, then every 3 months) for a total of 4 follow-up visits.

### Sample size 

To account for between-cluster variability, sample size calculations incorporated the coefficient of variation between pharmacies (*k*) [25, 26]. Assuming each cluster enrolls 95 (accounting for 15% attrition) AGYW and that 30% of participants achieve the primary outcome of PrEP persistence at 10 months (31) in the standard pharmacy PrEP model arm (control arm) arm with a coefficient of variation between clinics (*k*) of 0.05, 10 clusters per arm will give 90% power to detect a 15% absolute difference in proportions and a two-sided test with *α* = 0.05. Typical (*k*) values are 0.15 to 0.5 for rare binary outcomes and 0 to 0.05 for non-rare outcomes [25, 26]. Assuming 15% attrition, will enroll a final sample of 1900 ANC attendees at 20 sites (95 women per site; 950 women per arm).

### Recruitment 

AGYW who purchase a contraceptive method at a retail pharmacy study site will be recruited and enrolled. AGYW will be recruited into this trial following the purchase of contraception. Pharmacy staff will be aware of the study and asked to identify potentially eligible women at the point of contraceptive purchase. Pharmacy staff will explain the study to women who are potentially eligible and, if the woman expresses an interest in participating, will refer her to study staff. The study staff will then provide an in-depth explanation of the purpose and procedures of the study, in the language of the woman’s choosing, answer any questions the woman may have, and invite the woman to participate in the study. Participants will be informed that their participation is voluntary and that participation or nonparticipation in the study will in no way alter the nature of services that they receive at the pharmacy. If interested in participating, the study staff will screen the woman for eligibility, and, if eligible, the woman will provide written consent for their enrollment.

## Assignment of interventions: allocation

### Sequence generation 

Following pharmacy provider training, the study randomized 20 pharmacies 1:1 to either receive (*n* = 10) or not receive (*n* = 10) a nurse navigator arm to support PrEP delivery for AGYW. The pharmacy-level cluster randomization will be conducted using restricted randomization based on AGYW volume (i.e., monthly number of purchases made by AGYW clients) stratified by sub-county. Following study protocol training at an in-person event with staff from all 20 pharmacies, pharmacy staff were asked to select a ball with a random study arm assignment from a bag within each of the 10 groups to build camaraderie and demonstrate transparency of the randomization process.

### Concealment mechanism 

Since randomization occurred at the pharmacy level, it is impossible to blind study team members or participants to randomization assignments. However, procedures to minimize the influence of the unblinded nature of this study on outcomes are implemented. Randomization was only disclosed after all pharmacies were recruited and trained and signed agreements to participate in the study, following ethical approval. Ongoing data monitoring does not include information about study endpoints disaggregated by site or study arm. Only the study statistician will review data on study endpoints by study arm or facility.

### Implementation 

The randomization key of the data on study end points by study arm or pharmacy will be restricted to the data manager and statistician involved in the study.

## Assignment of interventions: blinding

### Who will be blinded 

N/A. The AGYW Pharmacy PrEP trial is an unblinded trial. Neither the participants nor the trial team will be blinded.

### Procedure for unblinding if needed 

N/A. The AGYW Pharmacy study is an open-label trial.

## Data collection and management

### Plans for assessment and collection of outcomes 

We will collect baseline data on demographic and behavioral characteristics, contraceptive history, education, marital status, income, relationship characteristics, HIV status and testing results, and clinical characteristics. At each visit, study staff will administer questionnaires to participants to assess sexual behavior, PrEP attitudes, use, and adherence. We will also measure IMB model-guided psychosocial moderating factors, including experience of IPV, symptoms of depression, socioeconomic status (SES), sexual and reproductive empowerment (SRE) and health, and PrEP use disclosure. We will assess SRE with the SRE-Kenya scale, which has been adapted and validated by our group for Kenyan AGYW. We will also capture PrEP-related side effects (e.g., nausea, headaches, dizziness) [27]. Fidelity will be monitored through QA/QC with direct observation of client-provider interactions using MEASURE Evaluation’s framework for monitoring quality of care in family planning services [28]. Hair samples (~ 50 strands) will be collected from all participants at every follow-up visit to objectively assess PrEP use and longitudinal analyses of PrEP use patterns.

HIV testing will be conducted at every visit per national guidelines. In a subset of four pharmacies, two from each study arm, we will conduct STI (for gonorrhea and chlamydia). The study utilizes the Cepheid Gene X-pert machine, a 4-module NAAT analyzer for real-time PCR testing of CT/NG and TV assays with expedited partner treatment for those with STI diagnoses to allow for analyses of STI incidence by arm.

### Plans to promote participant retention and complete follow-up 

Pharmacy providers and nurses will make follow-up reminders via phone calls and/or short messages. For the follow-up visits that occur in the clinic, participants will be reimbursed Kenyan shillings 300 (approximately $3 USD) for transportation costs per local cost of living and guidelines from the Kenya Medical Research Institute.

### Data management 

Research Electronic Data Capture (REDCap) hosted at University of Washington’s Institute of Translational Health Sciences is used for data collection and management. De-identified data will be uploaded to a secure server on a REDCap server accessible only to data managers, study coordinators, and principal investigators. REDCap’s capability for offline data entry will be used with password-protected tablets, with later use of a secure internet connection to upload data to local servers and a secure server. No participant personal identifiers will be entered in the REDCap database. All local databases will be secured with password-protected access systems. Forms, lists, logbooks, appointment books, and any other listings that link participant ID numbers to other identifying information will be stored in a separate, locked file in an area with limited access.

### Confidentiality 

Every effort will be made to protect participant privacy and confidentiality to the extent possible. Personal identifying information will be retained at the local study site and not forwarded to the University of Washington Coordinating Center. The site will use a standard operating procedure for confidentiality protection that reflects the input of study staff and community representatives to identify potential confidentiality issues and strategies to address them. All study-related information will be stored securely at the study site. All participant information will be stored in areas with limited access. Data collection, administrative forms, laboratory specimens, and other reports will be identified only by a coded number to maintain participant confidentiality. All records that contain names or other personal identifiers, such as locator forms and informed consent forms, will be stored separately from study records identified by code number. All local databases will be secured with password-protected access systems. Forms, lists, logbooks, appointment books, and any other listings that link participant ID numbers to other identifying information will be stored in a separate, locked file in an area with limited access. Only study staff will have access to electronic data via an encrypted server.

### Plans for collection, laboratory evaluation, and storage of biological specimens for genetic or molecular analysis in this trial/future use

Hair samples (~ 50 strands) will be collected from all participants at every follow-up visit to objectively assess PrEP use and longitudinal analyses of PrEP use patterns. A strong correlation between dosing frequency and ARV levels in the hair has been demonstrated [20] [1], and significant correlations were seen between the hair, plasma, peripheral blood mononuclear cells (PBMCs), and red blood cell (in dried blood spots, DBS) levels of PrEP drugs or metabolites [2, 3]. We will analyze hair samples to evaluate either TFV levels following daily oral PrEP use and DPV levels following DPV-VR use.

## Statistical methods

### Statistical methods for primary and secondary outcomes 

We will conduct an intention-to-treat analysis with the primary exposure of randomization arm (pharmacy-based PrEP delivery with nurse navigators vs. standard pharmacy-based PrEP delivery without nurse navigators) to test the hypotheses that incorporating nurse navigators within a pharmacy-based PrEP delivery model will improve PrEP initiation, persistence, and adherence among AGYW seeking contraception at retail pharmacies.

For analyses of our primary outcomes, we will use a log-binomial regression model to estimate relative risks and 95% CI. We will include any imbalanced baseline demographic, clinical, or behavioral characteristics between arms as independent variables in an adjusted model. If primary outcome data are missing or in cases of loss to follow-up, we will assume PrEP discontinuation/nonadherence to fit the most conservative models.

Secondary outcome analyses will include PrEP method selection, predictors of non-adherence, and STI incidence and cofactors. For data analysis approaches, we will use log-binomial regression with robust standard errors for the former two and Anderson-Gill survival analysis for the later. Exploratory analyses will include HIV incidence, quality of care, and contraceptive method mixed by randomization arms (Table 1). We will use survival analysis, generalized estimating equation models with logistic link, and robust standard errors and log binomial regression respectively.

### Interim analyses 

Our data and safety monitoring plan is our strategy for oversight and monitoring to ensure the safety of participants and the validity and integrity of data from the study. We will convene an external advisory panel (EAP) to review study aims, statistical analysis plan, and protocol. At annual EAP meetings, enrollment, retention, and pooled outcomes will be reviewed. The EAP can recommend suspension or termination of the study due to serious concerns about participants’ safety, inadequate performance, scientific policy developments that impact the study, or inadequate enrollment rates.

### Methods for additional analyses (e.g., subgroup analyses) 

Subgroup analyses will involve data from all AGYW at the enrollment visit to evaluate the effect of STI testing on PrEP initiation. We will also evaluate the association between offering STI testing at PrEP initiation and time-to-discontinuation using Cox proportional hazards models. We will have sufficient power to detect differences in the frequency of PrEP persistence of 14% or 60% between AGYW offered and not offered STI testing, assuming a 2-sided test with alpha = 0.05 and 80% power (*β* = 0.20). All models will use robust standard errors.

### Methods in analysis to handle protocol non-adherence and any statistical methods to handle missing data 

Study staff will be rigorously trained in PrEP guidelines, the study protocol, and standard operating procedures. A communication system will be in place whereby project staff and data entry staff will regularly discuss all aspects of data collection and problems that may arise. If primary outcome data are missing or in cases of loss to follow-up, we will assume PrEP discontinuation and nonadherence to fit the most conservative models.

### Plans to give access to the full protocol, participant level-data and statistical code 

Delinked and de-identified data will be made available by all participating institutions for public use 1 year after the data has been collected, scrutinized for errors, validated, and shared with any partners in data collection and the study results are published. More specifically, de-identified data will not include any information that can be used to identify an individual. Identifiers to be excluded include names, birth date, clinic visit date, biometric identifiers, and any other unique identifying number or code.

## Oversight and monitoring

### Composition of the coordinating center and trial steering committee

The entire project is jointly led by the University of Washington and Kenya Medical Research Institute- Kisumu to manage the coordination of site and laboratory-based activities. The coordinating center comprises of the principal investigators, project manager, research coordinator, study administrator, data manager, and laboratory manager. They ensure the day-to-day running of the trial and that it runs smoothly as per the protocol.

### Composition of the data monitoring committee, its role and reporting structure 

An external advisory panel (EAP) will be established to provide data monitoring and ensure the safety of study participants. Our data and safety monitoring plan (DSMP) is our strategy for oversight and monitoring to ensure the safety of participants and the validity and integrity of data from the study. The responsibility of the EAP will be to review recruitment, enrollment, and potential social harms at periodic intervals during the trial.

The study community outreach team will set up a community advisory board (CAB) in Kisumu County. The CAB communicates the community’s interests and concerns and advises on culturally appropriate ways to conduct the study. The study team will be giving regular updates on the study milestones and will incorporate CAB’s feedback.

### Adverse event reporting and harms 

The study team will monitor the conduct of the study in real-time through weekly summary reports of arms of accrual and baseline characteristics and quarterly reports of data pooled over treatment arms of data completeness, specimen collection, and adverse events (AEs). The study team will review individual participant-level safety data frequently to assess the relation of all reported AEs to study treatment. On a monthly basis, the study team will review summaries of premature study discontinuations and AEs. Notification to KEMRI ERC will be done within 48 h of identification of the SAE by the study team.

### Frequency and plans for auditing trial conduct 

The study team has mechanisms in place for internal audits that will be happening routinely to ensure data quality assurance. The data managers and study coordination will conduct periodic site visits to observe data recording and storage procedures and provide feedback. The ethical review or regulatory boards can also conduct random impromptu audits within the sites.

### Plans for communicating important protocol amendments to relevant parties (e.g., trial participants, ethical committees)

Protocol amendments will always be implemented once relevant ethical and regulatory approvals have been granted by the ethical review committee (ERC) and institutional review boards (IRB).

### Dissemination plans 

Upon completion of the study, written material summarizing the findings from this project will be made available to participants through the pharmacies. Additionally, the results will be presented to the clinical expert and policy advisors at the Kenya Ministry of Health and other officials. Additionally, the study will disseminate its findings by publication in peer-reviewed scientific journals, technical reports, policy briefs, study team meetings, and international scientific conferences.

## Discussion

Our results will have implications for other settings considering pharmacy-based PrEP delivery approaches for AGYW and will prime pharmacies to deliver novel PrEP agents in the pipeline (e.g., long-acting injectables and multi-purpose technologies). The study will inform nurse navigator approach for enhancing PrEP outcomes among AGYW and ultimately reducing HIV incidence in this important population disproportionately affected by HIV. Our study will advance PrEP implementation for AGYW and contribute insights on PrEP adherence and biomarkers (e.g., DPV-VR hair levels) that apply more broadly. In a subset of pharmacies, we will conduct STI (gonorrhea and chlamydia) screening and treatment including expedited partner treatment for those with STI diagnoses. This will be the first study to evaluate STI incidence among AGYW PrEP users in pharmacy setting, a population in which STIs have important implications, which can guide STI screening and treatment guidelines AGYW accessing PrEP outside of health facilities. If our nurse navigator approach positively affects non-HIV outcomes (e.g., increased access to desired contraceptive methods and counseling, sexual and reproductive empowerment), it could be readily integrated into other platforms (such as youth-friendly health clinics) aiming to improve SRH among AGYW.

In summary, the AGYW PrEP Pharmacy study may identify important information to guide the models for PrEP delivery which will benefit individuals in Kenya and other African countries with high HIV burden. The study will inform the policy to advance and incorporate community-based PrEP approaches for AGYW and ultimately contribute to minimizing the burden of HIV among AGYW in Kenya.

## Trial status

This publication was written regarding protocol version 1.1 from May 14, 2023. Recruitment commenced on May 17, 2023, and the first participant was enrolled on the same date. The recruitment phase is projected to continue until November 2024 with enrolled participants being followed for a duration of 10 months. Following the 10-month follow-up, participants will be referred to public health facilities for continuation of PrEP services.

## Data Availability

The investigators will have full access to the final dataset, and data required to support the protocol can be supplied on request. Data will be made available on open access with no identifiers included with the consent of study participants.

## Abbreviations

AGYW: Adolescent girls and young women
DPV-VR: Dapivirine vaginal ring
FP: Family planning
HIV: Human immunodeficiency virus
PrEP: Pre-exposure prophylaxis
